# The Placenta as an Immunological Environment

**DOI:** 10.3389/bjbs.2025.14910

**Published:** 2025-11-18

**Authors:** Fiona M. Menzies

**Affiliations:** School of Health and Life Sciences, University of the West of Scotland, Lanarkshire, United Kingdom

**Keywords:** placenta, immunomodulation, trophoblast, decidua, inflammation

## Abstract

In the UK, there are approximately 650,000 babies born each year. The pregnancy journey is not only unique to each woman, but for each individual pregnancy that may be experienced. Pregnancy complications, miscarriage, and stillbirths are still a huge problem with maternity services, highlighting the need for more research to understand the underlying causes, earlier detection or even prevention of conditions such as pre-eclampsia, gestational diabetes, restricted fetal growth and the impact of infection during pregnancy. One area of interest which transcends these conditions is the functioning of the placenta. The placenta is the lifeline for the fetus to the mother. It is a unique organ, crucial for survival, but also known to have impacts on the lifelong health of the fetus. Aberrant development, as well as *in utero* exposure to infections and environmental chemicals are known to have multiple impacts on the functioning of the placenta, and the fetus it supports. The placental environment is a fascinating organ to study with much still to be learned about its development, role in pregnancy complications, as well as its impact on long term offspring health. The placental environment is abundant with immune cells and mediators. There is a need within medical and biomedical practice for a good understanding of the complex relationship between immune cells, the decidua and placenta, and doing so will aid in development of better diagnostic tests and treatments for placenta-driven pregnancy complications and infections. This review will summarise the placenta as an immunological environment through description of key decidual immune cells, the expression of innate recognition receptors and it will provide an update on the placental immune response to infections of importance during pregnancy.

## Introduction

**FIGURE 1:**
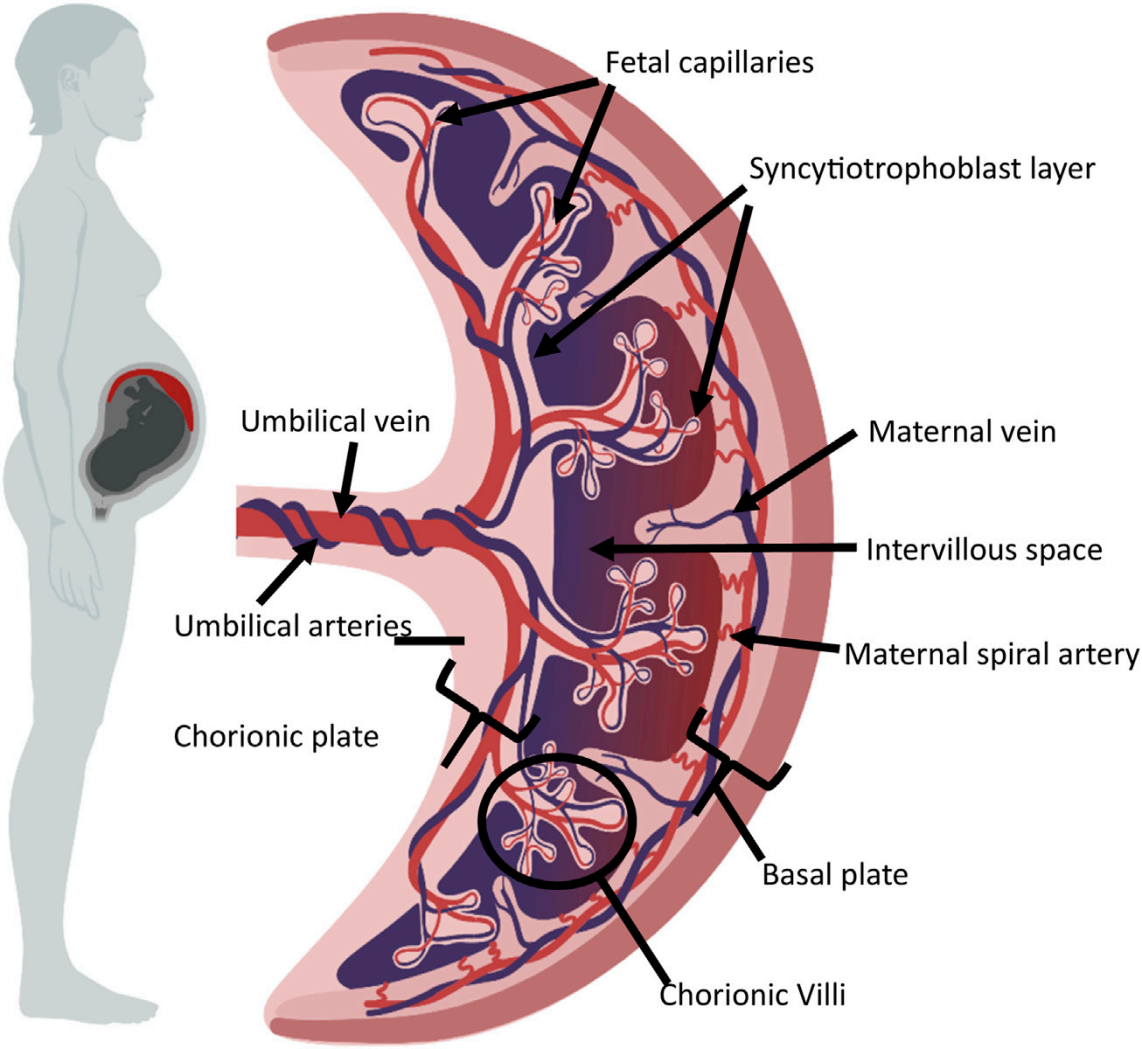
General overview of mature placental structure. The diagram shows the umbilical cord and placenta. Fetal capillaries are found within branches of chorionic villi, covered by the sycytiotrophoblast layer, and the underlying cytotrophoblast cells. The intervillous space separates the syncytiotrophoblast and maternal spiral arteries. This figure was created using BioRender.com.

The human placenta is a temporary, disc-shaped organ, weighing approximately 500g [1]. The placenta functions to support fetal development during pregnancy and is removed from the uterus after birth. It is multifunctional, supporting the transfer of oxygen and nutrients to the fetus, the removal of waste products, acting as an endocrine organ, and filtering fetal blood. Interestingly, the placenta is abundant in immune cells. Figure 1 provides a general overview of the structure of the mature placenta. The umbilical cord contains one umbilical vein and two umbilical arteries, carrying blood to and from the fetus, respectively. Within the placenta, fetal capillaries are found within branches of chorionic villi [2]. The epithelial covering of the villi is the syncytiotrophoblast layer, or syncytium, which is maintained by the fusion of underlying cytotrophoblast cells [2, 3]. The exchange of materials between fetus and mother takes place between the maternal blood within the intervillous space and the syncytiotrophoblast. Maternal spiral arteries deliver oxygen and nutrient-rich blood to the intervillous space, with maternal veins draining blood back to the maternal systemic circulation.

This review will consider our understanding of the immune function within the placenta, how immune cells are vital in the establishment of pregnancy, placentation and in the defence against pathogens.

## Placental Development

Placental development, or placentation, is a complex process. There are two key initial stages which must occur in tandem: firstly, successful blastocyst preparation, which involves hatching, apposition and attachment to the endometrial lining; and secondly, the creation of a uterine environment which is receptive to receive the blastocyst during the implantation phase. This latter process, decidualization, involves extensive changes within the endometrium to convert it into an environment capable of accepting the implanting conceptus. The blastocyst, which is derived from the fusion of a single ovum and sperm, is 50% antigenically foreign to the maternal immune system and requires a complex series of adaptations to ensure successful implantation. Indeed, the process of endometrial decidualization has been described as the primary driver of pregnancy health [4]. A delicate balance between pro-inflammatory and tolerance mechanisms is required, with inadequate endometrial immunomodulation linked to recurrent implantation failure [5, 6].

The blastocyst is formed around 5–6 days post-fertilization and in simplified terms, consists of an inner cell mass, which develops into the embryo, and an outer layer of trophoblast cells, which develops into the placenta. Around 7–8 days post-fertilisation, the blastocyst is ready to implant into the decidua. The decidua is abundant with immune cells, with leukocytes making up 30%–40% of all decidual cells in early pregnancy [7, 8]. This is a crucial consideration, as the cells of the blastocyst express paternal antigens, which have the potential to elicit a response from the cells of the maternal immune system. Therefore, understanding which cells are present, how their function is modulated, and indeed, how these cells contribute to the establishment of pregnancy furthers our ability to understand implantation failure and support embryo implantation during assisted conception treatment.

Placentation is usually established by around week 15, with three major trophoblast types described: cytotrophoblasts, extravillous cytotrophoblasts and syncytiotrophoblasts [9]. Much remains to be learned about this process, with single-cell RNA sequencing studies characterising the differentiation pathways between these trophoblastic cell types [10]. Generally, there is still much to be learned about the function and responses of the trophoblastic cell types within the placenta, as well as a need to fully characterize the function of the immune cell types, mediators and immunomodulators within the placenta. Indeed, Olmos-Ortiz and colleagues have reviewed how the placenta itself could be considered part of the innate immune system due to the plethora of components present [11].

## Immune Environment of the Placenta

### Innate Receptor Expression Within the Placenta

For the propagation of any innate immune response, there must first be recognition of the presence of a pathogen. Pattern recognition receptors (PRRs) are evolutionarily conserved germline-encoded receptors which can initiate responses upon recognition of molecular signatures of pathogens (pathogen associated molecular patterns, PAMPs) and damaged or dying cells (damage-associated molecular patterns, DAMPS) [12]. PRRs consists of a number of different receptor groups, including, but not limited to, Toll-Like Receptors (TLRs) [13], Nod-Like Receptors (NLRs) [14, 15], Scavenger Receptors [16] and RIG-like receptors (RLRs) [17].

Characterisation of PRR expression by trophoblastic cells remains a contentious and evolving area of placental immunology. Despite decades of research, consensus has yet to be reached regarding the precise expression profiles and functional relevance of PRRs, particularly TLRs, within the placenta. It is widely acknowledged that TLR expression within the placenta is not static but instead subject to temporal and spatial regulations [18], varying across gestational stages and trophoblast subtypes. This complexity is further compounded by the various methods used and often producing differing results; histology, qPCR, flow cytometry, single-cell RNA sequencing, for example. While qPCR provides quantitative gene expression data, it lacks spatial resolution; conversely, histological approaches can localise protein expression but may suffer from antibody specificity issues and subjective interpretation. Moreover, the advent of high-throughput technologies like single-cell RNA sequencing has begun to unravel previously unappreciated cellular heterogeneity within the trophoblast population, challenging earlier assumptions based on bulk tissue analysis [19–21]. Table 1 summarises key studies that have attempted to map PRR expression in placental trophoblasts, highlighting both converging and conflicting data.

**TABLE 1:** Summary of key studies exploring Pattern Recognition Receptor expression by human trophoblasts.

PRR	Expression pattern	Placenta stage/type of trophoblasts (detection method)	References
TLR1	First trimester trophoblasts	Primary trophoblasts (qPCR)	[147]
TLR2	Extravillous trophoblasts	First trimester primary trophoblasts (IHC)	[148]
	Villous/intermediate trophoblasts	Term placenta (IHC)	[149]
	First trimester trophoblasts	Primary trophoblasts (qPCR)	[147]
TLR3	First trimester trophoblasts	Primary trophoblasts (qPCR)	[147]
	Cytotrophoblasts and syncytiotrophoblasts	First trimester cytotrophoblasts, third trimester syncytiotrophoblasts (mRNA expression, IHC of placental explants)	[150]
TLR4	Extravillous trophoblasts	First trimester primary trophoblasts (IHC)	[148]
	Villous/intermediate trophoblasts	Term placenta (IHC)	[149]
	Extravillous trophoblasts/intermediate trophoblasts, villous Hofbauer cells	Second, third trimester (IHC)	[151]
	First trimester trophoblasts	Primary trophoblasts (qPCR)	[147]
	Cytotrophoblasts and syncytiotrophoblasts	First trimester cytotrophoblasts, third trimester syncytiotrophoblasts (mRNA expression, IHC of placental explants)	[150]
TLR5	First trimester trophoblasts	Primary trophoblasts (qPCR)	[147]
TLR6	First trimester trophoblasts	Primary trophoblasts (qPCR)	[147]
TLR7	First trimester trophoblasts	Primary trophoblasts (qPCR)	[147]
TLR8	First trimester trophoblasts	Primary trophoblasts (qPCR)	[147]
TLR9	First trimester trophoblasts	Primary trophoblasts (qPCR)	[147]
TLR10	Villous and extravillous trophoblasts	First trimester and term (IHC, Western blotting, qPCR)	[152]
	First trimester trophoblasts	Primary trophoblasts (qPCR)	[147]
NOD1	Extravillous trophoblasts	First trimester primary (IHC, Western blotting, qPCR)	[153]
NOD2	Extravillous trophoblasts	First trimester primary (IHC, Western blotting, qPCR)	[153]
RIG-1	Syncytiotrophoblasts	Term (IHC)	[29]
	Chorionic villi & decidua	Third trimester, mRNA and protein expression (qPCR, Western blotting)	[154]
	Trophoblasts	Term placenta (IHC)	[155]
MDA5	Chorionic villi & decidua	Third trimester, mRNA and protein expression (qPCR, Western blotting)	[154]
	Trophoblasts and villous stroma	Term placenta (IHC)	[155]

TLR, Toll Like Receptor; NOD, nucleotide oligomerization domain; RIG, retinoic acid-inducible gene; MDA, melanoma differentiation-associated protein; qPCR, quantitative polymerase chain reaction; IHC, immunohistochemistry.

A recent comprehensive review by Motomura and colleagues [22] has attempted to synthesise these disparate findings, offering a more cohesive narrative on PRR expression in the human placenta. Importantly, it extends beyond mere characterisation to interrogate the functional implications of PRRs in pregnancy complications such as preeclampsia, intrauterine growth restriction, and preterm birth. This shift from descriptive to mechanistic inquiry marks a critical evolution in the field, underscoring the need for integrative approaches that combine molecular profiling with functional assays and clinical correlation. Future research must prioritise standardisation of methodologies and embrace systems-level approaches to fully elucidate the immunological landscape of the placenta and its role in maternal-fetal health.

Aberrant expression of PRRs has emerged as a pivotal factor in the pathogenesis of pre-eclampsia, a multifaceted syndrome characterized by a convergence of vascular, immunological, and placental dysfunctions [23, 24]. While the precise etiological sequence remains under investigation, it is increasingly evident that there are underlying immune modulations [25], which either contribute to the development of pre-eclampsia, or are a consequence of pre-eclampsia. Notably, pre-eclamptic placentas exhibit increased TLR4 expression [26], implicating heightened TLR-mediated signalling in the amplification of local inflammation, oxidative stress and poor extravillous cytotrophoblast invasion [24, 27, 28]. These disruptions compromise spiral artery remodeling, a hallmark of placental insufficiency in pre-eclampsia. Emerging evidence suggests that the immune landscape of the placenta differs between early and late onset pre-eclampsia, with distinct TLR expression profiles potentially reflecting divergent pathogenic trajectories. Despite the extensive focus on TLRs and their role in pre-eclampsia, the potential role of other types of PRR in pre-eclampsia are now emerging. For example, it has been shown, in contrast to the expression pattern of TLR4, that retinoic acid-inducible gene I (RIG-I), a cytosolic RNA sensor of the RIG-I-like receptor (RLR) family, exhibits markedly reduced expression in placentas of both late and onset pre-eclampsia [29]. This downregulation may signify impaired antiviral defense or altered immune tolerance at the maternal-fetal interface, further contributing to the pro-inflammatory milieu. The differential expression patterns of PRRs underscore the complexity of immune dysregulation in pre-eclampsia and highlight potential avenues for biomarker development and targeted immunomodulatory therapies.

Another pregnancy complication of particular interest is gestational diabetes mellitus (GDM). In the UK, approximately 5% of pregnant women have either pre-existing diabetes or GDM with around 87.5% of those diagnosed during pregnancy having GDM [30]. Early diagnosis and treatment of GDM are critical for the safe progression of the pregnancy and delivery, as well the future health of mother and baby. GDM is known to be associated with placental dysfunction; in most cases, the condition resolves after delivery of the placenta. Central to its pathophysiology is a state of chronic low-grade inflammation and immune dysregulation at the maternal-fetal interface, which compromises placental integrity and nutrient exchange.

Mechanistically, GDM placentas exhibit upregulated expression of TLR4, the adaptor protein MyD88 and the transcription factor NF-kB [31–34]. This signalling axis is a canonical pathway in innate immunity, where TLR4 recruits MyD88 to initiate downstream signalling cascades. MyD88 serves as a critical scaffold, facilitating the activation of interleukin-1 receptor-associated kinases (IRAKs) and TNF receptor-associated factor 6 (TRAF6), culminating in the nuclear translocation of NF-κB [35]. Once activated, NF-κB drives the transcription of pro-inflammatory cytokines (for example TNF-α, IL-6, IL-1β), chemokines, and adhesion molecules, fostering a pro-inflammatory milieu within the placenta [36]. This environment disrupts trophoblast differentiation, impairs spiral artery remodelling, and alters insulin signalling pathways, further exacerbating maternal insulin resistance [37]. Moreover, heightened TLR4/MyD88/NF-κB signalling may contribute to endothelial dysfunction and oxidative stress, both of which are implicated in adverse pregnancy outcomes such as fetal macrosomia, preterm birth, and increased risk of type 2 diabetes in offspring [38, 39]. Clinically, these insights underscore the importance of early GDM screening and targeted interventions, not only to manage glycaemic control but also to modulate inflammatory pathways. Emerging therapeutic strategies, including dietary modulation, anti-inflammatory agents, and microbiome-targeted therapies, may hold promise in attenuating placental inflammation and improving maternal-fetal outcomes.

### The Decidual Environment

The decidua is an immune cell-rich site and during the establishment of pregnancy, the populations of immune cells and their functions adapt to support the environment. These cells include uterine Natural Killer (uNK) cells, macrophages (including Hofbauer cells), T cells and dendritic cells (DCs) (Table 2). Functional adaptations of immune cells (compared to function in the non-pregnant situation) are observed during early pregnancy, alongside the unique expression of Human Leukocyte Antigen (HLA) molecules by fetal extravillous cytotrophoblast.

**TABLE 2:** Summary of decidual immune cells.

Trimester	Cell Type	Role	References
1	uNK	uNK1 and uNK2 accumulate in the first trimester	[56, 57]
		Trophoblast-uNK cell interactions lead to a suppression on activation	[55]
	Macrophages	M1 (inflammatory) type dominates	[67, 156]
		Hofbauer cells produce factors promoting angiogenesis	[72]
	T cells	Th1 cells dominate during the implantation window	[88]
		CD8^+^ Tc cells make up 45% of the decidual leukocyte population	[157]
2	uNK	Loss of trophoblast-uNK interaction Activating receptor expression increased	[55]
	Macrophages	M2 (anti-inflammatory) type dominates	[67, 156]
	T cells	Th2 cells and tregs dominate	[88]
		CD8^+^ T cell numbers increase in the decidua throughout pregnancy, they exhibit a silenced phenotype (compared to peripheral cells)	[96]
3	uNK	Increased degranulation response, less recognition of HLA-C	[158]
	Macrophages	M1 (inflammatory) type dominates	[67, 156]
	T cells	High number of Tfh cells	[159]
		CD8^+^ T cell numbers increase in the decidua throughout pregnancy, they exhibit a silenced phenotype (compared to peripheral cells)	[96]

uNK, uterine Natural Killer cell; Th, T helper; Tc, Cytotoxic T cell; HLA, Human Leukocyte Antigen; Tfh, T follicular helper.

Unlike most somatic cells, extravillous trophoblasts do not express classical MHC class I molecules HLA-A, HLA-B, nor the class II HLA-D molecule. Instead, they selectively express HLA-C, HLA-E, HLA-F and HLA-G [40], which are pivotal in shaping the maternal-fetal immune interface. HLA-C, like -E, -F and -G is expressed on the surface of extravillous cytotrophoblast [41], HLA-E and -F show strong expression in the first trimester [40]. HLA-E is frequently co-expressed with HLA-G [42] and binds to NKG2A and NKG2C receptors on uNK cells and γδT cells, modulating their activity. HLA-F, although less understood, fluctuates during the menstrual cycle and peaks during the implantation window. It correlates with CD56^+^ NK cell density and may influence implantation success through receptor interactions and genetic polymorphisms that affect its expression.

HLA-G exhibits robust expression throughout gestation and has emerged as a central regulator of immune tolerance. It exists in both membrane-bound and soluble forms and is robustly expressed throughout gestation, not only by extravillous trophoblasts but also by Hofbauer cells, endothelial cells of chorionic villi, amniotic cells, and the umbilical cord epithelium [40, 43]. Recent findings show that HLA-G interacts with inhibitory receptors such as ILT2, ILT4, and KIR2DL4 on uNK cells, leading to suppression of cytotoxic activity and promotion of growth factor secretion that supports placental development [44–46]. Moreover, HLA-G has been implicated in activating senescence signaling pathways in NK cells, which contributes to spiral artery remodeling, a process essential for adequate placental perfusion [47–49].

Importantly, recent research highlights that decidual γδT cells, a less-studied immune subset, express receptors for HLA-E and HLA-G and produce both angiogenic factors (e.g., G-CSF, FGF2) and cytotoxic mediators (e.g., Granulysin, IFN-γ) [50, 51], suggesting a dual role in placental development and pathogen defense. These findings challenge the traditional view of immune suppression during pregnancy and suggest a more nuanced model of immune modulation and functional specialization. The selective expression of non-classical HLA molecules by EVTs is not merely a passive shield against maternal immune attack. It actively orchestrates a complex interplay of immune tolerance, vascular remodeling, and tissue growth, processes that are increasingly understood through advances in molecular immunology and reproductive biology.

### Uterine NK (uNK) Cells

In the first trimester decidua, uNK cells are abundant. uNK cells differ significantly from peripheral blood NK cells. The majority of uNK cells are defined as CD56^bright^CD16^−^CD3^−^ [52–54]. Throughout pregnancy the function of these cells change. Interestingly, uNK cells accumulate near to extravillous cytotrophoblast in the first trimester, and less so in the second trimester [55]. Until fairly recently, it was thought uNK cells were a single population of cells. It has now been recognised that there are three distinct population [10], generally named uNK1, uNK2 and uNK3. These subpopulations differ on the basis of their chemokine and immunomodulatory properties and are present at different times of gestation. uNK1 and uNK2 are most abundant in the first trimester, however by the third trimester, uNK3 are the dominant population [56, 57]. Given this pattern, it is not surprising that uNK1 and uNK2 are considered to play important roles in implantation [57], in particular they are considered to be important in the process of spiral artery remodelling [54]. Indeed, uNK cells make up 30% of the immune cell population during the implantation window, and then in early pregnancy, they make up 70%–80% of the immune cell population in the decidua [54, 58, 59].

In addition to these pregnancy-supportive roles, uNK cells also play roles in resistance to infection and immune tolerance of the fetus. uNK cells lack cytotoxicity [60, 61] and the inhibition of degranulation is mediated through the Gal-9/Tim-3 signalling cascade [62]. The interaction between uNK cells and trophoblast cells is key for development of immune tolerance. It is hypothesized that these cellular interactions lead to a reduction in uNK cell function [55]. Interestingly, recent meta-analysis investigating the relationship between uNK cells and recurrent miscarriage and recurrent implantation failure has challenged earlier assumptions that elevated uNK cell numbers are directly pathogenic. While no consistent correlation was found between uNK cell quantity and pregnancy outcomes, a more nuanced picture has emerged regarding uNK cell phenotype and receptor expression. Several studies report that women with recurrent miscarriage have uNK cells with lower expression of inhibitory receptors such as KIR2DL1, KIR2DL4 and NKG2A [63]. These receptors are critical for recognising non-classical HLA (e.g., HLA-G and HLA-E) expressed by extravillous trophoblasts, and their engagement typically suppresses cytotoxic responses while promoting vascular remodeling and immune tolerance.

Diminished expression of these inhibitory receptors may impair the immunomodulatory dialogue between uNK cells and trophoblasts, leading to inadequate spiral artery remodeling, heightened local inflammation, or inappropriate immune activation against fetal antigens. This receptor-level dysfunction underscores that functional competence, rather than cell abundance, is a more reliable marker of uNK cell contribution to pregnancy success. It also highlights the importance of maternal-fetal HLA-KIR compatibility, which has been linked to preeclampsia and fetal growth restriction in other studies [64, 65]. Clinically, these findings suggest that immunophenotyping of uNK cells, especially receptor profiling, may offer more predictive value than simple cell counts in assessing reproductive risk. It also opens avenues for targeted immunotherapies aimed at restoring receptor expression or modulating NK cell function in women with unexplained recurrent miscarriage or recurrent implantation failure.

### Macrophages

Macrophages are vital players at the maternal-fetal interface in the induction of tolerance, defence against pathogens and establishment of pregnancy. As key players of the innate immune system, they have crucial roles in the initial stages of immune responses, however our understanding of these cells has grown in recent years, and we now have a better appreciation of the complexity of this cell types, including the existence of subtypes, dependent on the local environment and cytokine milieu in which these cells find themselves. Traditionally classified into pro-inflammatory M1 and anti-inflammatory M2 subsets, this binary framework has proven overly simplistic. Recent advances in single-cell RNA sequencing have revealed a spectrum of macrophage phenotypes, shaped by the dynamic cytokine milieu and tissue-specific signals of the decidua [10, 66]. These studies show that decidual macrophages co-express markers of both M1 and M2 states, suggesting functional plasticity rather than fixed polarisation. This heterogeneity enables macrophages to simultaneously support trophoblast invasion, regulate inflammation, and maintain immune homeostasis, underscoring their nuanced role in reproductive immunology.

Within the decidua, the balance across the M1 and M2 phenotype changes throughout pregnancy, with inflammatory M1 dominating the early stages to aid in the process of implantation, M2 dominating the mid stages to ensure maintenance of pregnancy, and then a return to M1 dominance at the point of parturition, where inflammation helps drive uterine contractions [67]. It is likely that these changes are driven by changes in pregnancy hormones and their impact on the production on granulocyte macrophage colony stimulating factor (GM-CSF) and macrophage colony stimulating factor (MCSF) [67] which drives M1 and M2, respectively. The importance of the balance between M1 and M2 is illustrated by studies which show that higher numbers of M1 macrophages in the decidua are associated with miscarriage [68, 69].

In the context of the placenta, we also have to mention the importance of Hofbauer cells; macrophages (of fetal origin) located around the placental villi [70]. These cells can be detected in the placenta as early as 4 weeks post-conception [71]. Studies of the first trimester placenta has revealed that Hofbauer cells have a unique phenotype compared to other macrophages; they do not express HLA-DR and they produce a number of factors (for example, IL-8 and MMP-9) which may contribute to placentation, mainly angiogenesis and spiral artery remodelling [72].

### Dendritic Cells (DCs)

Dendritic cells (DCs) are professional antigen presenting cells, which are viewed as crucial initiators of immune responses through sampling antigen and presenting to T cells within lymph nodes. Early flow cytometric studies have shown that within the decidua, dendritic cells are located throughout both the decidua basalis and decidua parietalis [73]. Generally, the decidua contains a greater number of conventional DCs, and less plasmacytoid DCs than peripheral blood [74]. One way in which DCs contribute to the tolerance of paternal antigens, and therefore the fetus, is by entrapment within the decidua [75]. That is, DCs within this tissue do not leave to present to T cells at lymph nodes and initiate immune responses.

Building on the current understanding of decidual DCs, recent findings have begun to challenge and refine long-held assumptions about their role in pregnancy. While entrapment within the decidua has been proposed as a mechanism for promoting tolerance, emerging evidence suggests that this may not be a universal feature, particularly in pathological contexts such as recurrent spontaneous abortion, where DCs exhibit a more activated phenotype. For example, recent studies have shown that in some cases of recurrent spontaneous abortion, women have DCs with increased expression of MHC Class II, CD80 and CD86 [76]. This raises important questions about the plasticity and regulation of DC function *in situ*. Moreover, the discovery that DCs contribute to stromal cell differentiation in murine models expands their role beyond immune modulation, suggesting a dual function in both immunological and structural aspects of placental development [77]. However, the precise signalling pathways mediating these interactions, such as the involvement of TGF-β, Wnt, or Notch signalling, remain poorly defined [78, 79]. There is also a lack of consensus on how DC subsets are influenced by the decidual microenvironment, and whether these changes are reversible or developmentally programmed. These gaps underscore the need for integrative approaches, including spatial transcriptomics and functional assays, to dissect the context-dependent roles of DCs and their contribution to both tolerance and tissue remodelling.

### T cells

There are numerous types of T cells with differing functions and identified by their unique surface expression of certain markers, transcription factors and cytokine production. Cytotoxic T cells (Tc) are defined as CD3^+^CD8^+^ and T helper (Th) cells are identified generally as CD3^+^CD4^+^ and can be further subcategorised into Th1, Th2, Th17, T regulatory (Treg), Th9 and Follicular T helper (Thf) cells.

Th cells undoubtedly play important roles during the preimplantation phase, with the balance between the different Th subsets crucial for early pregnancy success. Implantation is described as an inflammatory process, and with the dominance of inflammatory M1 macrophages at the site of implantation, a dominance in Th1 type cells is also observed. Th1 cells typically produce cytokines such as tumour necrosis factor (TNF)-a and interferon (IFN)-g. While these are potent inflammatory cytokines, studies have shown during implantation they contribute to the control of trophoblast invasion and mobility [80, 81], however aberrant systemic expression of these Th1-associated cytokines is associated with implantation failure and miscarriage [82, 83]. Interestingly, Th17 cells follow a similar pattern to Th1 cells. During early pregnancy, Th17 cells are recruited to the decidua by decidual stromal cells through the release of the chemokine CCL2 [84]. It has been hypothesized that they function to inhibit trophoblast apoptosis [84]. As with circulating Th1 cells and cytokines, Th17 cells and IL-17 have been linked with PE [85, 86]. Research suggests that pre-eclampsia is associated with an imbalance between Th17 cells and regulatory T cells (Tregs), leading to excessive immune activation at the maternal-fetal interface. Increased levels of memory-like Th17 cells is found in pre-eclamptic placentas, suggesting their involvement in disease progression [87].

The maintenance of pregnancy is associated with a dominance in Th2 cells and Tregs [88–90] and a downmodulation in Th17 cells. Th2 cells are characterised by the production of the cytokines IL-3, -4, -5 and -13, and at the maternal-fetal interface, these cytokines are associated with pregnancy success [91]. These Th2 cytokines, including IL-4, IL-5, IL-10, and IL-13, contribute to immune modulation by reducing cytotoxic T-cell activity and promoting regulatory mechanisms that support fetal survival. Additionally, Th2-driven immunity facilitates the development of maternal-fetal tolerance through interactions with regulatory T cells (Tregs).

Tregs are indepensable for the maintenance of pregnancy, primarily through their role in dampening the maternal immune response against fetal antigens. This balanced immune adaptation ensures successful implantation and placental development. Seminal studies using murine models have demonstrated that depletion of Tregs during early gestation leads to fetal resorption and pregnancy failure, underscoring their crucial function for the maintenance of pregnancy through controlling immune reactivity at the maternal-fetal interface [92, 93]. Indeed, more recent studies have shown that the absence of Tregs leads to rejection of the fetus. More recent investigations have employed single-cell RNA sequencing to reveal that decidual Tregs undergo transcriptional reprogramming in response to local cues, acquiring a tissue-adapted effector phenotype [64, 94]. For instance, decidual Tregs upregulate genes associated with tissue residency (e.g., CD69, CXCR3) and immune regulation (e.g., IL10, CTLA4), distinguishing them from their peripheral counterparts [94]. Collectively, findings from murine depletion models, human tissue profiling, and transcriptomic analyses highlight the dynamic and context-dependent nature of Tregs in pregnancy. Their ability to adapt phenotypically and functionally to the decidual niche is central to preventing fetal rejection and ensuring gestational success.

Given their cytotoxic role in the immune response, it is interesting to consider that CD8^+^ Tc cells are the most abundant T cells at the decidua [95]. Extravillous cytotrophoblasts do not express HLA-A and HLA-B, however they do express HLA-C which is a potential candidate for CD8^+^ Tc cell recognition. However, in successful pregnancies, this recognition and downstream cytotoxic action does not occur [95]. Analysis of the CD8^+^ Tc cell populations within the decidua have shown that compared with peripheral CD8^+^ Tc cells, the decidua contains populations that have a phenotype resembling effector memory cells, which only partial effector functions [96].

## Placental Exosomes as Immunomodulators

Despite the first report of placental exosomes being published some time ago [97], this is an area of placental research which has gained momentum in recent years. Placental exosomes are being investigated as biomarkers of maternal diseases as reviewed in several publications [98–102] and for their potential use as non-invasive diagnostic tools.

Exosomes are extracellular vesicles that originate from the endosome of a cell, tend to be around 40–160 nm in diameter [99] and can carry different molecule types such as proteins, lipids, mRNA, non-coding RNAs and DNA fragments. In doing so, exosomes act as a transport mechanism for genetic and protein information between cells.

Placental exosomes are released through a multistep process. Exosomes originate within endosomes, where they accumulate in multivesicular bodies (MVBs). These MVBs contain small vesicles that eventually become exosomes. Once matured, MVBs fuse with the placental cell membrane, releasing their contents into the extracellular space. The release of exosomes is influenced by factors such as oxygen levels, glucose concentration, and maternal stress [103]. These conditions can alter the number and composition of exosomes secreted. Exosomes are continually shed from the syncytiotrophoblast into the mother’s bloodstream throughout pregnancy [104–106], although their production is significantly increased in the first trimester [107, 108]. Once in maternal circulation, placental exosomes interact with immune and vascular cells, influencing maternal physiology and fetal development.

**FIGURE 2:**
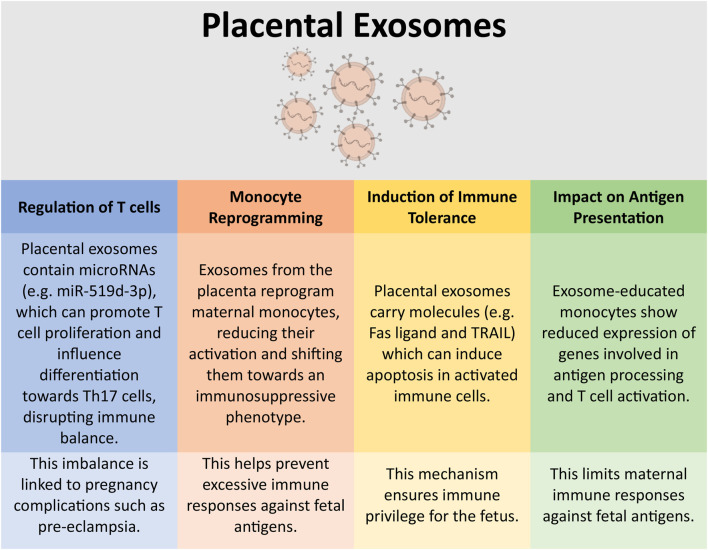
Immunomodulation by placental exosomes. Placental-derived exosomes can have a number of impacts on the immune system. This figure highlights some of these key immunological interactions [117, 118, 146]. This figure was created in part using BioRender.com.

Placental exosomes mediate immune tolerance during pregnancy [109–112] through localised immune modulation within the uterine and placental environments. They do this through a number of mechanisms, some of which are summarised in Figure 2. Placental exosomes can promote the secretion of IFN-γ and VEGF by uNK cells via HLA-E secretion [113], reduce the expression of the NKG2D activating receptor on cytotoxic NK cells [114], and modulating the differentiation, activation and polarisation of decidual macrophages [115, 116]. Finally, placental exosomes have been shown to downmodulate T cell proliferation and cytotoxity, and driving Treg differentiation [117]; placental exosomes target and alters the activity of monocytes to influence these impacts on T cells [109, 118].

Placental exosomes are increasingly recognized as key players in pregnancy complications. These extracellular vesicles carry bioactive molecules that influence maternal physiology, and their altered composition can signal pathological conditions. For example, exosomes from pre-eclamptic placentas show dysregulated microRNA profiles, affecting vascular function and immune responses [104]. Placental exosomes have also been implicated in the pathogenesis of GDM. Placental exosomes in GDM pregnancies contain molecules that alter insulin sensitivity, contributing to metabolic imbalances [119]. Exosomes from compromised placentas may impair nutrient transport, affecting fetal development [120] and changes in exosomal cargo can influence inflammatory pathways, potentially triggering premature labour [121]. These findings suggest that placental exosomes could serve as biomarkers for early detection of pregnancy complications and may even be targeted for therapeutic interventions [122].

## Placental Exposure to Infection

Infections are a threat to the survival of both mother and fetus and there are a number of ways in which an infection could cause damage, by impacting the mother’s health, the fetus’ health or the functioning of the placenta. The placenta utilizes mechanisms, where possible, to limit the vertical transmission of pathogen from mother to fetus. There are, however, some infections which can bypass the placental barrier to cause congenital infection. These are the TORCH infections: *Toxoplasma gondii*, other, rubella virus, cytomegalovirus, herpes simplex virus. These infections induce immune responses within the placenta which are often damaging to it, leading to placental dysfunction in addition to vertical transmission of the pathogen. In the UK, the TORCH infections are diagnosed using a combination of serological tests and direct pathogen detection methods; (1) Blood tests check for antibodies (IgM and IgG) against TORCH pathogens, helping determine past exposure or active infection, (2) polymerase chain reaction (PCR) assays detect viral DNA or RNA, particularly useful for CMV and HSV, (3) imaging and amniotic fluid analysis help assess fetal health and detect congenital infections, and (4) Infants suspected of congenital infection undergo blood tests, hearing assessments, and ophthalmologic exams.

### *Toxoplasma gondii* (*T. gondii*)

*T. gondii* infection in the immunocompetent is usually asymptomatic, but presents with flu-like symptoms in ∼20% of cases and is self-limiting [123]. Congenital toxoplasmosis is caused by the transmission of live parasites through the placenta to the fetus. This tends to occur only when primary infection is acquired during pregnancy; prior infections generates sufficient control of the infection to prevent transmission. Control of *T. gondii* infection involves a robust Th1 type response [124], which is in contrast with the Th2-type environment that dominates within the placenta. This therefore means the placenta presents an environment in which the parasite will thrive. Generation of a Th1 type immune response to control the parasite is detrimental to the placenta. One way in which *T. gondii* tachyzoites gain entry to the decidua is by the “trojan horse” theory where the parasite “hides” in immune cells [125]. It is also thought that tachyzoites infect invading extravillous cytotrophoblast and the syncytiotrophoblast layer [126].

In the context of UK clinical practice, congenital toxoplasmosis remains a significant concern due to its potential for severe fetal outcomes, including hydrocephalus, chorioretinitis, and intracranial calcifications. Routine antenatal screening for *T. gondii* is not currently implemented in the UK, unlike in some European countries such as France and Austria, where early detection allows for timely intervention. Diagnosis typically relies on serological testing following clinical suspicion or ultrasound findings suggestive of fetal infection. When maternal infection is confirmed during pregnancy, spiramycin is often initiated to reduce transplacental transmission, although its efficacy is limited to early gestation and it does not treat established fetal infection. In cases where fetal infection is confirmed, usually via amniocentesis and PCR, pyrimethamine and sulfadiazine are considered, despite their teratogenic and hematologic risks [102, 127]. These limitations underscore the need for improved diagnostic tools and safer therapeutic options. Furthermore, the immunological paradox of requiring a Th1 response to control *T. gondii,* despite its incompatibility with the placental Th2-dominant environment, poses a challenge for immunomodulatory strategies. Greater awareness among clinicians is essential for timely diagnosis and management.

### Rubella

Rubella is a rare viral infection which causes mild or no symptoms in most people, however, if contracted during pregnancy, can lead to miscarriage, stillbirth or development of fetal defects. Early studies have shown that rubella infection during pregnancy presented a number of impacts on the placenta, including a reduction in placental weight, villitis and disruption to the villous architecture [128]. In 90% of cases, Congenital Rubella Syndrome occurs during the first 8 weeks of pregnancy, with ∼30% occurring in the second trimester [129]. Infection of trophoblast cells is associated with production of the type I interferons [130], and the proinflammatory chemokine CCL5 [131] which can be detrimental to the placental environment through driving inflammation as well as trophoblast migration and invasion, potentially disrupting placental development [132].

In UK clinical practice, rubella is now exceedingly rare due to the success of the national MMR (measles, mumps, rubella) vaccination programme, which offers protection to children and is routinely checked in women of childbearing age [133]. However, sporadic cases still occur, particularly among individuals born outside the UK or those with incomplete vaccination records. When rubella infection is suspected during pregnancy, urgent serological testing is performed to assess maternal immunity and confirm recent infection. If primary infection is confirmed in early pregnancy, referral to fetal medicine specialists is essential due to the high risk of Congenital Rubella Syndrome, especially in the first trimester. Ultrasound monitoring may reveal signs of fetal compromise, and parents are counselled regarding prognosis and management options. The immunopathological findings, such as placental inflammation and disrupted trophoblast function, highlight the importance of preconception vaccination and robust antenatal screening protocols. Public health efforts continue to focus on maintaining high vaccine uptake and identifying at-risk populations.

### Human Cytomegalovirus

As was described for *T. gondii* and Rubella, human cytomegalovirus can be harmless to those who are immunocompetent, but dangerous for those who are immunocompromised and in infants. As reported by Fisher and colleagues, the placenta is not an effective barrier to cytomegalovirus infection [134]. Placental cytotrophoblasts and syncytiotrophoblasts possess receptors, such as integrin α1β1 and integrin αVβ3 which facilitate the transmission of cytomegalovirus through the placenta [135]. Cytomegalovirus is never eliminated by the body; it establishes latency within cells of the myeloid lineage such as CD14^+^ monocytes and can be reactivated during inflammatory responses [136]. Studies have shown that cytomegalovirus leads to the downregulation of HLA-G expression by cytotrophoblast cells [134, 137]. It is hypothesised that this is one way in which the immune system tries to respond to the virus, by downregulating the tolerogenic mechanism which protects the placenta.

In UK clinical practice, congenital cytomegalovirus infection is the most common viral cause of neurodevelopmental disability [138], yet routine antenatal screening is not currently implemented. Diagnosis typically arises following ultrasound findings suggestive of fetal infection such as ventriculomegaly, intrauterine growth restriction, or echogenic bowel, or through targeted maternal serology and PCR testing. When primary cytomegalovirus infection is confirmed during pregnancy, management is complex due to the lack of licensed antiviral treatments proven safe and effective for fetal use. Valaciclovir has shown some promise in reducing viral load and transmission risk, but its use remains off-label and limited to specialist settings [139]. The immunological findings, such as viral exploitation of placental integrins [135] and downregulation of HLA-G [134, 140], highlight the challenges in balancing maternal immune tolerance with antiviral defence. These mechanisms may help explain why cytomegalovirus can cross the placenta even in immunocompetent individuals. Greater awareness among clinicians, improved diagnostic pathways, and ongoing research into maternal immunomodulation are essential to reduce the burden of congenital cytomegalovirus in the UK.

### Herpes Simplex Virus (HSV)

Herpes Simplex Virus (HSV) is a double-stranded DNA virus that is responsible for genital and oral herpes. There are two main viral serotypes, HSV-1 and HSV-2, with the latter described as the most commonly sexually transmitted infection [141]. Transmission of HSV can occur through the vagina/cervix or via the placenta, and can occur at any stage of pregnancy [142]. As reported by Deftereou and colleagues [142] the mechanism by which the virus transmits through the placenta is debatable, however, it has been reported that damage to the syncytiotrophoblast layer is required to allow viral entry [126]. Placental infection results in chronic inflammation, with histopathological studies showing lymphocytic infiltration into the villous tree [143].

In UK clinical practice, neonatal herpes simplex virus (HSV) infection is rare but carries a high risk of morbidity and mortality, particularly when acquired intrapartum or via transplacental transmission. Routine antenatal screening for HSV is not currently recommended, but clinical vigilance is essential, especially in women with a history of genital herpes or presenting with active lesions during pregnancy. Management involves suppressive antiviral therapy, typically acyclovir, from 36 weeks gestation to reduce viral shedding and the risk of transmission during vaginal delivery [144]. In cases of primary infection in late pregnancy, or active lesions at term, elective caesarean section is considered to minimise neonatal exposure. The histopathological findings of placental inflammation and syncytiotrophoblast disruption highlight the importance of early recognition and intervention, particularly in symptomatic women or those with known HSV seroconversion during pregnancy. Neonatal HSV infection, when suspected, prompts urgent virological testing and initiation of intravenous aciclovir, with multidisciplinary input from obstetrics, neonatology, and infectious disease teams to guide care and reduce long-term neurological sequelae [145].

## Conclusion

The placenta is an extraordinarily complex and dynamic organ that performs a multitude of essential functions throughout gestation. Beyond its well-known roles in nutrient transfer, gas exchange, and hormone production, the placenta serves as a critical immunological interface between the mother and fetus. One of its most remarkable capabilities is its ability to simultaneously promote immune tolerance toward the semi-allogeneic fetus (bearing paternal antigens) while maintaining robust defences against invading pathogens. This immunological balancing act has profound implications for antenatal care, particularly in the context of infection screening, immunological monitoring, and the management of pregnancy-related complications such as preeclampsia, intrauterine growth restriction, and recurrent miscarriage.

To achieve this balance, the placenta must develop in a manner that supports fetal growth and survival while evading maternal immune rejection. This involves a highly orchestrated interplay of cellular and molecular mechanisms, including the recruitment and regulation of immune cells within the decidua; the specialized maternal tissue at the maternal-fetal interface. This review has highlighted key populations of decidual immune cells, such as uNK cells, macrophages, and Tregs, which are present in high abundance and play pivotal roles in modulating immune responses. Additionally, the expression of PRRs, including TLRs and NOD-like receptors, enables the placenta to detect and respond to microbial threats, thereby contributing to its innate immune surveillance system.

This review also provided an updated overview of infections that pose significant risks during pregnancy and considered how placental immune responses can influence vertical transmission and fetal outcomes. As research continues to elucidate the molecular pathways by which the placenta modulates immune responses to paternal antigens and microbial stimuli, new opportunities are emerging to translate these insights into clinical practice. For instance, a deeper understanding of placental immunology could inform the development of targeted fertility treatments, optimize protocols for assisted reproductive technologies, and refine immunosuppressive strategies in organ transplantation by leveraging mechanisms of maternal-fetal tolerance.
